# Hydrocephalus as an Initial Presentation of Myelin Oligodendrocyte Glycoprotein (MOG) Antibody-Associated Disease: A Case Report and Review of Tumor-Mimicking Demyelination

**DOI:** 10.7759/cureus.89246

**Published:** 2025-08-02

**Authors:** Makoto Ishii, Satoko Arai, Ryotaro Ikeguchi, Yuko Shimizu, Kenichi Todo

**Affiliations:** 1 Department of Neurology, Tokyo Women’s Medical University, Tokyo, JPN

**Keywords:** brain biopsy, brain histopathology, brain tumor, deep gray matter, demyelinating disease, germinoma, hydrocephalus, myelin oligodendrocyte glycoprotein antibody-associated disease, third ventriculostomy, tumefactive lesion

## Abstract

Myelin oligodendrocyte glycoprotein (MOG) antibody-associated disease (MOGAD) is a demyelinating disease of the central nervous system (CNS) that manifests as optic neuritis, transverse myelitis, acute disseminated encephalomyelitis, and cortical encephalitis. Some patients with MOGAD present with tumor-like brain lesions. However, hydrocephalus as an initial presentation is rare. We present the case of a 23-year-old Japanese man with an acute onset of headache, nausea, and diplopia, who was initially suspected of having a germinoma but was later diagnosed with MOGAD. Brain magnetic resonance imaging (MRI) revealed a tumor-like lesion with hyperintensity on fluid-attenuated inversion recovery (FLAIR) imaging and contrast enhancement on T1-weighted postcontrast images, extending from the midbrain to the thalamus with obstructive hydrocephalus. Neuroendoscopic third ventriculostomy and brain biopsy were performed. Histopathological analyses revealed demyelination, perivascular lymphocytic infiltration, and MOG loss. MOG antibody tests were positive, confirming MOGAD. The patient was treated with pulse steroid therapy (methylprednisolone 1,000 mg/day) and seven sessions of plasmapheresis, resulting in significant neurological improvement. He was discharged approximately two months after symptom onset, and at the six-month follow-up from discharge, he remained relapse-free with only mild diplopia. In this case, early diagnosis via pathological brain analysis and anti-MOG antibody testing allowed for timely treatment. We emphasize the importance of including MOGAD in the diagnostic workup of tumor-mimicking CNS lesions that can cause hydrocephalus.

## Introduction

Myelin oligodendrocyte glycoprotein (MOG) antibody-associated disease (MOGAD) is an inflammatory demyelinating disorder of the central nervous system (CNS) that typically presents with optic neuritis, transverse myelitis, acute disseminated encephalomyelitis (ADEM), and cerebral cortical encephalitis [1,2]. MOGAD is increasingly recognized as a distinct disease entity different from multiple sclerosis (MS) and aquaporin-4 (AQP4)-positive neuromyelitis optica spectrum disorder (NMOSD) and is more prevalent among children and young adults. While some patients with MOGAD develop tumor-like lesions in the brain, hydrocephalus as an initial manifestation is exceedingly rare [3,4]. The mechanism by which hydrocephalus may arise is thought to involve mass effects. Germinomas, on the other hand, are primary intracranial germ cell tumors that typically affect male adolescents and commonly arise in the pineal or suprasellar regions [5,6]. These tumors frequently present with obstructive hydrocephalus and demonstrate homogeneous enhancement on contrast-enhanced magnetic resonance imaging (MRI). When tumor markers such as β-human chorionic gonadotropin (βHCG) and placental alkaline phosphatase (PLAP) are elevated and imaging is typical, germinomas are often treated empirically with radiotherapy without histological confirmation. This case report presents a diagnostically challenging case of MOGAD in a young adult who initially presented with obstructive hydrocephalus and an imaging profile mimicking a germinoma. We emphasize the importance of considering demyelinating diseases in the differential diagnosis of tumor-like brainstem lesions and highlight the potential role of early antibody testing.

## Case presentation

**Figure 1:**
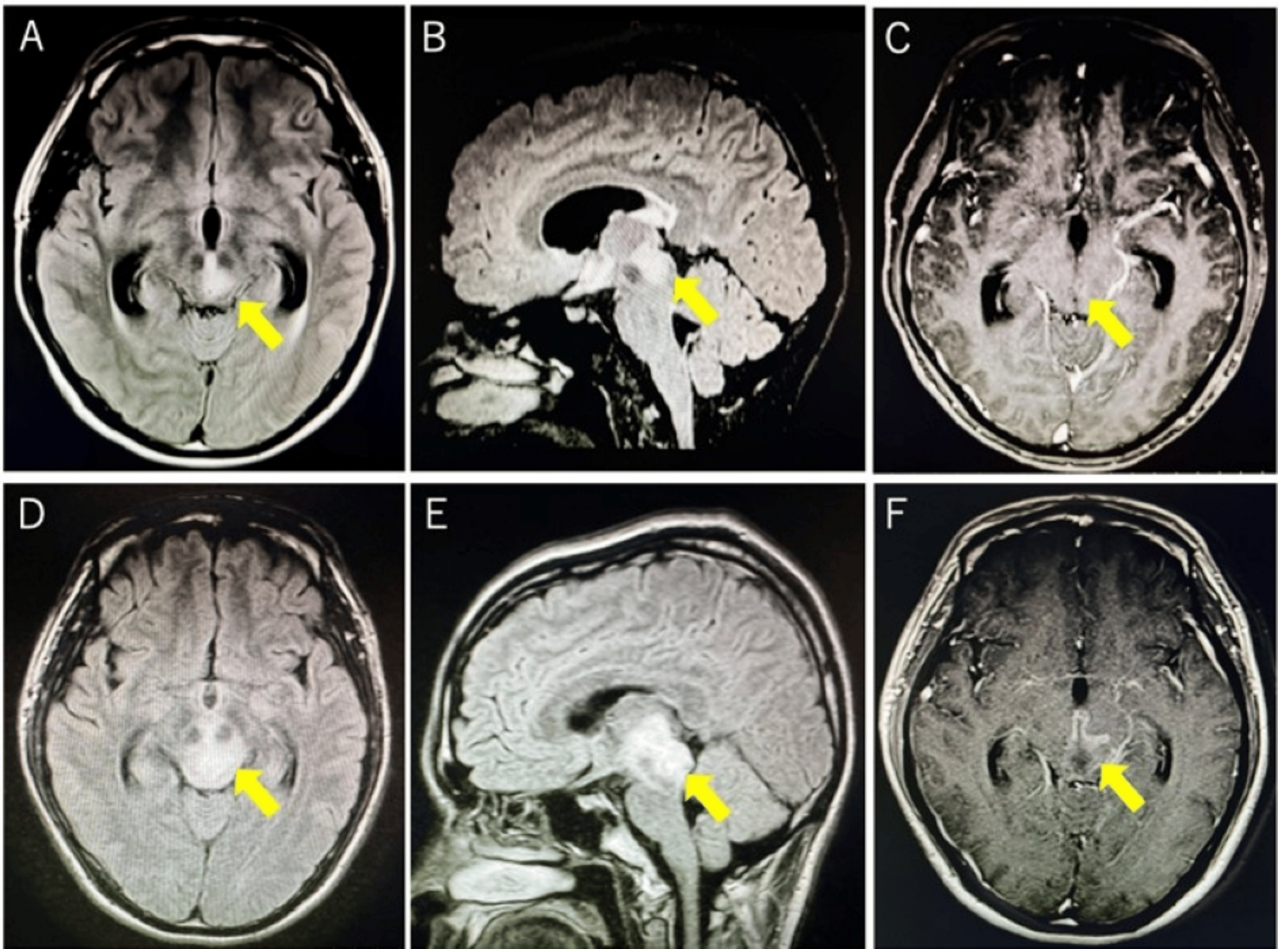
Brain MRI at initial presentation and postoperative day nine MRI findings at initial presentation (A-C). Axial (A) and sagittal (B) FLAIR images show a hyperintense lesion extending from the left thalamus to the left midbrain, accompanied by obstructive hydrocephalus with dilation of the third and lateral ventricles. (C) Axial T1-weighted postcontrast image reveals mild heterogeneous enhancement. Postoperative brain MRI findings on hospital day nine (D-F). Axial (D) and sagittal (E) FLAIR images show significant progression of the midbrain lesion, indicating increased inflammatory activity, whereas hydrocephalus has resolved. (F) Axial T1-weighted postcontrast image demonstrates enhancement of the lesion. MRI: magnetic resonance imaging; FLAIR: fluid-attenuated inversion recovery

A 23-year-old previously healthy Japanese man presented with an acute onset of a diffuse dull headache and nausea and developed pan-directional diplopia on the following day. His medical, family, and social histories were unremarkable, and he had no recent history of infection or vaccination. On day six after symptom onset, he was admitted to our hospital. The patient was afebrile, with stable vital signs: body temperature 36.3°C, pulse rate 57 bpm, and blood pressure 110/53 mmHg. Neurological examination revealed markedly asymmetrical severe ptosis (more pronounced on the left); bilateral limitations of adduction, elevation, and depression; and absence of pupillary light reflexes, suggesting oculomotor nerve impairment. No sensory, motor, or coordination deficits were observed. However, he exhibited mild drowsiness; he was arousable to verbal stimuli and followed simple commands, though spontaneous speech was reduced. Fluid-attenuated inversion recovery (FLAIR) MRI of the brain revealed a hyperintense lesion extending from the left thalamus to the midbrain with mild contrast enhancement. The lesion had a maximum diameter of 10 mm and was associated with obstructive hydrocephalus characterized by dilation of the third and lateral ventricles (Figures 1A-1C).

**Figure 2:**
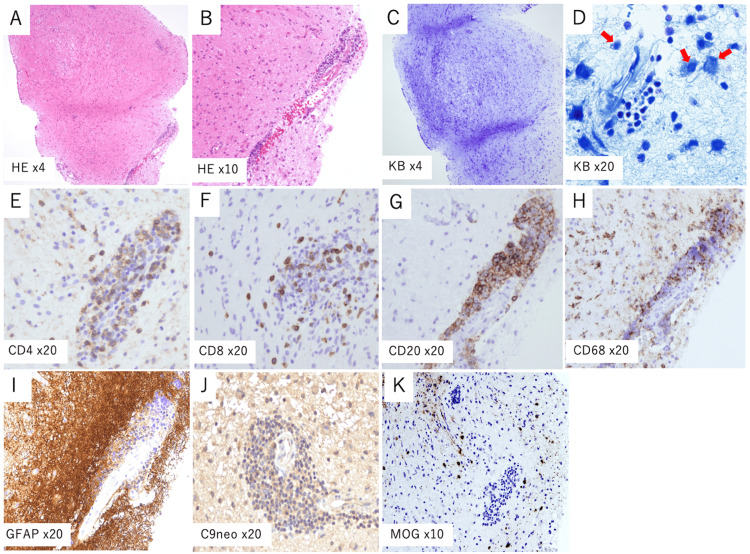
Histopathological findings of the biopsied midbrain tissue (A, B) Hematoxylin and eosin (HE) staining reveals perivascular lymphocytic infiltration. (C, D) Klüver–Barrera (KB) staining demonstrates demyelination accompanied by foamy macrophages and myelin debris phagocytosis (D, arrows). (E, F) CD4 and CD8 staining reveals a predominance of CD4+ T-cells over CD8+ T-cells. (G) CD20 staining indicates moderate B-cell infiltration, which appears more prominent compared to CD4+ and CD8+ T-cells. (H) CD68+ macrophages are also observed in the perivascular lesion. (I) GFAP staining demonstrates upregulation, suggesting pronounced astrocytic activation. (J) C9neo staining demonstrates complement deposition. (K) MOG staining shows a marked loss of MOG in the perivascular lesion. GFAP: glial fibrillary acidic protein; MOG: myelin oligodendrocyte glycoprotein

Methionine positron emission tomography (PET) showed mild uptake within the lesion (tumor-to-normal tissue count ratio = 2.0), suggesting the possibility of a low-grade neoplasm. Cerebrospinal fluid (CSF) analysis revealed a slightly elevated cell count (8 cells/μL, lymphocyte predominant), a protein level of 40 mg/dL (upper limit: 40 mg/dL), and negative tumor markers for germ cell tumors (βHCG and PLAP). The initial opening pressure was 80 mmH_2_O. Routine laboratory tests including serum collagen disease and vasculitis markers and infectious markers, such as tuberculosis, were normal, and serum anti-AQP4 antibody was absent. Due to worsening headache and nausea over the course of nine days after admission, along with clinical suspicion of a tumor, a neuroendoscopic third ventriculostomy and biopsy of the midbrain tegmentum were performed on hospital day nine. Brain histopathological examination revealed perivascular lymphocytic infiltration, foamy macrophages, and demyelination, with no evidence of neoplastic cells (Figures 2A-2D).

**Figure 3:**
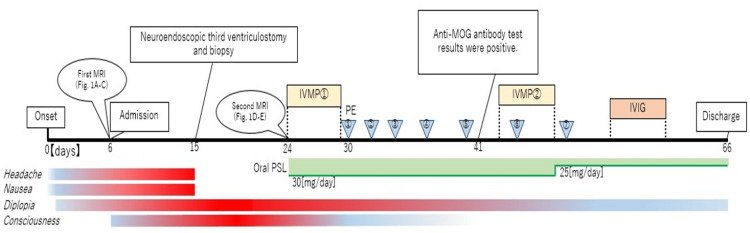
Clinical course with key interventions and symptoms The horizontal bars represent the time course of symptoms (headache, nausea, diplopia, and consciousness disturbance). The intensity of the red color reflects symptom severity. Key clinical events, including neuroendoscopic third ventriculostomy and biopsy (day 9) and confirmation of anti-myelin oligodendrocyte glycoprotein (MOG) antibody positivity (day 24), are annotated along the timeline. Immunotherapies included two courses of intravenous methylprednisolone pulse (IVMP: methylprednisolone 1,000 mg/day for five days), intravenous immunoglobulin (IVIG: 400 mg/kg/day for five days), plasma exchange (PE), and tapering oral prednisolone (PSL). MRI: magnetic resonance imaging

Postoperatively, the patient showed improvement in headache and nausea. However, his level of consciousness further declined daily, and oculomotor nerve function worsened. MRI revealed further enlargement of the lesion with ring enhancement on postoperative day nine, although the hydrocephalus had improved (Figures 1D-1F). Subsequent CSF analysis revealed a mononuclear pleocytosis (77.7/μL), increased interleukin-6 (318 pg/mL; normal <4.3 pg/mL), and myelin basic protein (>500 pg/dL; normal <103 pg/mL). The IgG index was not elevated, and oligoclonal bands were absent. CSF cultures were negative for bacteria and acid-fast bacilli. Brain immunohistochemistry revealed the predominance of CD4+ T-cells over CD8+ T-cells, infiltration of CD20+ B-cells, and moderate complement deposition. Notably, MOG immunostaining was reduced, whereas glial fibrillary acidic protein was upregulated (Figures 2E-2K). Based on these findings, pulse steroid therapy (methylprednisolone 1,000 mg/day) was initiated on postoperative day nine, followed by maintenance therapy with oral prednisolone (30 mg/day). However, as the MRI showed continued lesion progression, a second course of pulse steroids, intravenous immunoglobulin (IVIG; 400 mg/kg/day for five days), and plasma exchange (PE) was initiated. After seven sessions of PE, the patient’s neurological symptoms improved significantly. Mild diplopia persisted, and the patient was discharged. Both serum and CSF tests for MOG antibodies using a live cell-based assay (IgG1) on postoperative day 26 yielded positive results. The serum MOG-IgG was positive at a dilution of 1:4, indicating a high-titer result, confirming the diagnosis of MOGAD (Figure 3).

To prevent relapse, additional IVIG therapy was administered one month after discharge, and no regular maintenance IVIG therapy (e.g., every 4-6 weeks) was performed thereafter. At the six-month follow-up after discharge, the patient’s diplopia had improved. Serum testing revealed that MOG antibodies were undetectable. Oral prednisolone was tapered to 15 mg/day, and the patient has remained relapse-free since discharge.

## Discussion

MOGAD is a recently identified inflammatory and demyelinating disease. Recently, it has been reported that some people with MOGAD present with brain tumor-like lesions [3,4]. These lesions are often misinterpreted as brain tumors because of their imaging characteristics, such as mass effect, edema, and contrast enhancement. In addition to MOGAD, various demyelinating and inflammatory diseases can present with tumor-like brain lesions, including MS, NMOSD, ADEM, and CNS vasculitis [3].

In the present case, a germinoma was initially suspected based on the imaging findings (location of the lesion) and patient demographics. Germinomas predominantly affect young males, particularly those aged 10-30 years, with approximately 80% of the cases occurring in pediatric populations [5]. These tumors most commonly arise in the pineal or suprasellar regions, accounting for over 90% of cases, and frequently lead to obstructive hydrocephalus [6]. Tumors occurring in the thalamus and basal ganglia account for approximately 4%-20% of cases [7]. Differentiating between germinoma and MOGAD can be challenging because factors, such as age, sex, and brain MRI findings, are not clearly distinguished. Elevated germinoma biomarkers (βHCG and PLAP) in the serum and CSF serve as useful diagnostic tools [8,9]. In germinoma, when tumor markers are elevated and imaging reveals lesions in typical locations such as the pineal or suprasellar regions, radiation therapy is often initiated empirically without histological confirmation. However, in the present case, the germinoma biomarkers did not increase. The lesion was located in the midbrain tegmentum, a deep-seated region of the brain where the proximity to vital brainstem structures rendered stereotactic brain biopsy technically and safely challenging. However, due to worsening symptoms of intracranial hypertension secondary to hydrocephalus, emergent surgical intervention was required. The decision to perform a neuroendoscopic third ventriculostomy in combination with a diagnostic brain biopsy during this procedure significantly contributed to establishing the diagnosis and enabling early therapeutic intervention.

Currently, there is a lack of published data regarding methionine PET uptake patterns in MOGAD. In our case, the lesion demonstrated mild methionine uptake (tumor-to-normal tissue count ratio = 2.0), which raised concern for a low-grade neoplasm. However, this case suggests that demyelinating lesions associated with MOGAD can show mild methionine uptake, potentially mimicking neoplastic processes. Clinicians should be cautious in interpreting methionine PET results in young patients with tumefactive lesions, particularly when other findings are atypical for tumors.

Demyelinating diseases, including MOGAD, rarely present with hydrocephalus compared to tumors. This may be related to the lower frequency of mass effect in demyelinating lesions. However, in some cases with strong inflammatory responses, demyelinating lesions can exhibit significant edema and mass effect, potentially leading to CSF flow obstruction. There have been several reports of inflammatory demyelinating diseases that present with hydrocephalus, including MS, NMOSD, and MOGAD (Table 1). Although the exact mechanism remains unclear, hydrocephalus in demyelinating diseases such as MS and NMOSD has been attributed to various factors, including mass effect and inflammation-related CSF flow impairment [10-14].

**Table 1:** Literature review of reported cases of hydrocephalus in MS, NMOSD, and MOGAD CSF: cerebrospinal fluid; EVD: external ventricular drain; F: female; LETM: longitudinally extensive transverse myelitis; M: male; MOGAD: myelin oligodendrocyte glycoprotein antibody-associated disease; MS: multiple sclerosis; N/A: not available; NMOSD: neuromyelitis optica spectrum disorder; OCB: oligoclonal band; PE: plasma exchange; VP shunt: ventriculoperitoneal shunt All NMOSD cases were positive for aquaporin-4 antibodies.

	Štourač et al. (2021) [10]	Gratton and Mora (2013) [13]	Clardy et al. (2014) [11]			Close et al. (2019) [14]	Ronhatgi et al. (2023) [12]	Sinha et al. (2022) [15]	Present case
Disease	MS	NMOSD	NMOSD			NMOSD	NMOSD	MOGAD	MOGAD
Age	29	54	40	19	36	35	32	10	23
Sex	F	F	F	F	F	F	F	F	M
Initial clinical symptom	Headache, cognitive, gait problems	Unresponsive	Headache, nausea, vomiting	Headache, nausea, vertigo	Neck pain, confusion, quadriparesis	Headache, somnolence, fatigue, lethargy	Headache, transient visual obscurations, gait imbalance	Vomiting, somnolence, aphasia, hemiparesis	Headache, nausea, diplopia
CSF findings	OCB (+)	N/A	N/A	N/A	N/A	N/A	No pleocytosis	206 cells/μL OCB (-)	62 cells/μL OCB (-)
Region	Septum pellucidum region	Peri-callosal white matter	Superior cerebellar peduncle	Superior cerebellar peduncle	N/A	Periventricular region	Periventricular and periaqueductal regions	Periventricular and centrum semiovale lesion extended into the brainstem	Thalamus, midbrain
Hydrocephalus	Obstructive	Non-obstructive	Obstructive	Obstructive	Obstructive	Non-obstructive	Obstructive	Obstructive	Obstructive
Treatment	Natalizumab, VP shunt	EVD, PE, cyclophosphamide	Shunt	VP shunt	Corticosteroids, shunt	Ventriculostomy, VP shunt	VP shunt, corticosteroids, azathioprine	Corticosteroids, hemicraniectomy, EVD, PE, cyclophosphamide	Third ventriculostomy, corticosteroids, PE
Prognosis	Died from meningitis post-shunt	Alert and appropriately interactive	Required multiple shunt revisions	Undergone successful third ventriculostomy	No LETM recurrence over 18 years	Returned to neurological baseline	Vision loss recurred 8 months later	Speaking fully and mild right lower spasticity	Mild diplopia persisted

Sinha et al. reported a case of MOGAD in a 10-year-old girl who presented with hydrocephalus. The patient required an externalized ventricular drain [15]. In this case, the initial presentation involved subcortical white matter extending from the left frontal to the parietal lobes, which expanded over the course of a week. The lesion exhibited tumefactive characteristics accompanied by a midline shift and obstructive hydrocephalus. Hydrocephalus is a rare but reported complication in MOGAD. The mechanism may involve inflammatory lesions causing mass effect or direct obstruction of CSF pathways, particularly when lesions are located near the mesencephalic aqueduct or third ventricle. In our case, the lesion extended from the thalamus to the midbrain tegmentum, adjacent to the cerebral aqueduct, which contributed to obstructive hydrocephalus. The frequency of tumefactive brain lesions in MOGAD is higher than that in MS or NMOSD [3,4]. Therefore, the incidence of hydrocephalus in patients with MOGAD may be higher than that in patients with MS or NMOSD.

The pathological features of MOGAD typically include perivascular lymphocytic infiltration, myelin phagocytosis by macrophages, and loss of MOG with relatively preserved myelin basic protein, myelin-associated glycoprotein, and neurofilament. Infiltrating lymphocytes generally show a predominance of CD4+ T-cells over CD8+ T- and B-cells. Complement deposition is generally mild compared to that in NMOSD [4,16]. In the present case, the pathological findings were consistent with common MOGAD features, such as perivascular lymphocytic infiltration, demyelination, and gliosis. Mild complement deposition and MOG loss were also observed. Notably, the present case showed a predominance of B-cell infiltration. Previously, we reported two cases of tumor-like MOGAD with predominant B-cell infiltration [4]. Commonalities between these two cases and the present case include the young age of the patients (19 and 15 years in the previously reported cases and 23 years in the present case), suggesting that B-cell-predominant infiltration might be a feature of MOGAD in younger individuals. Additionally, both previously reported cases exhibited large lesions in the deep gray matter, aligning with the present case, where the lesion extended from the midbrain to the thalamus. These observations highlight the importance of considering MOGAD, especially in young patients with deep gray matter lesions. Furthermore, ADEM is more common in younger individuals with MOGAD [17], and B-cell-predominant lymphocytic infiltration may be a characteristic feature of ADEM-type MOGAD in young-onset cases.

Collectively, differentiating hydrocephalus in demyelinating diseases, including MOGAD, from brain tumors, such as germinomas, remains a significant diagnostic challenge. Both conditions share overlapping brain imaging features, including mass effects and contrast enhancement. In addition, CSF analysis often provides non-specific findings, offering limited diagnostic value. Furthermore, germinomas and MOGAD share similar epidemiological features and predominantly affect young patients. In the present case, the early detection of MOG antibodies in the serum and CSF was crucial for establishing the diagnosis, underscoring the importance of MOG antibody testing in the differentiation of young patients presenting with tumor-like findings. Limitations of this study include its nature as a single-case report, which limits the generalizability of the findings. In particular, the diagnostic value of methionine PET uptake and the significance of B-cell-predominant infiltration in young-onset MOGAD require further investigation in larger cohorts.

## Conclusions

MOGAD should be considered in the differential diagnosis of tumefactive brain lesions, particularly in young patients presenting with obstructive hydrocephalus and negative tumor markers. While rare, hydrocephalus may be an under-recognized manifestation of MOGAD when lesions involve periaqueductal or brainstem regions. Early recognition through imaging, pathological analysis, and MOG antibody testing is crucial to avoid misdiagnosis and ensure timely immunotherapy. Additionally, B-cell-dominant infiltration may be a characteristic feature of young-onset MOGAD, although further studies are needed. This report highlights the importance of including MOGAD in the diagnostic workup of tumor-mimicking CNS lesions.
